# Advances in genetic developmental and epileptic encephalopathies with movement disorders

**DOI:** 10.1186/s42494-024-00194-z

**Published:** 2025-02-03

**Authors:** Meng Yuan, Xiaoqian Wang, Zuozhen Yang, Huan Luo, Jing Gan, Rong Luo

**Affiliations:** 1 Department of Pediatrics, West China Second University Hospital, Sichuan University, Chengdu, 610041 China; 2 Department of Pediatrics, Yibin Second People’s Hospital, Yibin, 644000 China; 3 Cipher Gene LLC, Beijing, 100089 China

**Keywords:** Developmental and epileptic encephalopathies, Genetic, Movement disorders

## Abstract

Genetic developmental and epileptic encephalopathies (DEE) are often associated with movement disorders. Accurate identification and classification of movement disorders are essential for management of these diseases. In this review, we describe the characteristics of various movement disorders associated with DEE and summarize the distribution of common DEE-related gene mutations reported in previous studies, aiming to provide references for the diagnosis and treatment of these disorders.

**Supplementary Information:**

The online version contains supplementary material available at 10.1186/s42494-024-00194-z.

## Background

In 2017, the International League Against Epilepsy (ILAE) further classified epileptic encephalopathies and introduced the concept of developmental and epileptic encephalopathies (DEE). This term refers to a group of epileptic encephalopathies in which the encephalopathy state of the patient is caused by the combined effects of underlying developmental factors and epileptic abnormalities. DEE typically manifests during the neonatal period, infancy, or childhood and is marked by significantly abnormal electroencephalograms (EEGs) and frequent pharmacoresistance [1]. DEE exhibits clinical and genetic heterogeneous and can have multiple potential causes, including brain structural abnormalities, genetic metabolic disorders, and monogenic genetic diseases [2–4]. With advancements of genetic testing technology, especially the wide application of next-generation sequencing technology in the clinic, 118 DEE-associated mutated genes are now included in the Online Mendelian Inheritance in Man (OMIM) database. In published literature, 40–50% of DEE have been found to be associated with genetic variants, involving over 800 mutated genes [5–7]. In the diagnosis and treatment of genetic DEE, we often focus on the seizures, EEG findings, and imaging characteristics of the patients, but they are often accompanied various forms of movement disorders [8–11]. Genetic DEE-associated movement disorders have specific characteristics, making it important to understand these specific characteristics in the diagnosis and treatment of patients [10, 11]. Therefore, after reviewing recent literature on genetic DEE with movement disorders, we summarize the characteristics of the movement disorders observed in these patients.

## Possible mechanisms of genetic DEE-associated movement disorders

The mechanisms of genetic DEE-associated movement disorders are currently unclear and may involve multiple factors, including:

1. The protein functions affected by the mutated genes may be widely present and/or indispensable throughout the central nervous system. Such gene mutations may lead to widespread neuronal dysfunctions, clinically manifesting as a combination of several neurological features, including global developmental delay, seizures, and movement disorders. These observations have been made in cases with mutations in *WDR45*, *EPG5*, *SNX14*, *UBE3A*, and *HACE1 * [12–14].
2. Mutations in genes that regulate synaptic functions, including those involved in G-protein-coupled signal transduction, neurotransmitter transmission, and synaptic vesicle cycling, may impact diverse neuronal subtypes within cortical and subcortical networks, resulting in seizures and movement disorders [8].
3. Some gene mutations cause structural alterations in the brain. When these change affect structures that are involved in both motor and epileptogenic networks, they lead to seizures and movement disorders, such as mutations in *SLC9A6*, *FOXG1*, *SPTAN1*, *WDR45*, and *SNX14 * [14–18].

## Diagnostic strategies for genetic DEE-associated movement disorders

The classification of movement disorders is based on clinical characteristics, which are used to distinguish between hyperkinetic and hypokinetic categories [19]. Hyperkinetic movement disorders presented with abnormal involuntary movements, which may be regular and rhythmic (e.g., tremor), more persistent and patterned (e.g., dystonia), brief and random (e.g., chorea), or tic-like and temporarily suppressible (e.g., tics) [19]. These disorders include tics, dystonia, stereotypies, chorea, tremor, and myoclonus, with tics being the most common hyperkinetic movement disorder in children [19]. Hypokinetic movement disorders are often associated with muscle rigidity, postural instability, and reduced spontaneous movement [19]. Accurate classification and diagnosis of movement disorder is more difficult in patients with complex neurological conditions [20]. Movement disorders include dystonia, stereotypies, choreoathetosis, tremor, myoclonus, and hypokinesia are often associated with genetic DEE, and the same individual is often accompanied by common of different movement disorders [10, 11].

Accurate classification and diagnosis of these movement disorders in patient with genetic DEE require detailed history of the patient and the necessary auxiliary examinations. Video recording of the patient’s movement disorders can be used in retrospective analysis, especially for paroxysmal disorders; early imaging studies are also necessary, which not only providing diagnostic clues but also excludes structural brain abnormalities caused by acquired factors. In addition, EEG should be used to differentiate paroxysmal movement disorders from seizures, and genetic metabolic disorders should be excluded when necessary [8].

## Different types of movement disorders

### Stereotypies

Stereotypies are simple, repetitive movements that can be voluntarily suppressed, manifesting as repetitive behaviors such as chewing, rocking, spinning, or touching, and are often paroxysmal [19, 21]. Stereotypies can be distinguished from seizures using EEG. While stereotypies can occur in normal individuals, they are more prevalent in patients with schizophrenia, intellectual disabilities, and autism spectrum disorders [19]. The mechanisms underlying stereotypies are not fully understood, but they are believed to involve psychological and biological factors, including genetic, brain structural and functional, and neurochemical factors [22]. Psychological hypotheses suggest that stereotypies may compensate for external sensory deficiencies, redirect excessive action capacity, substitute for imagined activities, or be performed as part of compulsive or anxiety-related behaviors [23]. Neuroimaging studies have identified dysfunction in the cortico-striato-thalamo-cortical circuits as being associated with stereotypies, with the neurochemical mechanisms possibly involving dopaminergic, GABAergic, and cholinergic systems [22]. Genetically, stereotypies are a prominent movement disorder in Rett syndrome, caused by mutations in the *MECP2* gene [24]. Stereotypies are also common in patients with genetic DEE, particularly when mutations involve genes related to transcription factors and transcriptional regulation (e.g., *CHD2*, *TBL1XR1*, *MECP2*, *MEF2C*, *BRAT1*, *CDKL5*, *MBD5*, *HNRNPU*, *PURA*, *SETD5*) and synaptic membrane function (e.g., *STXBP1*, *DNM1*, *DNM1L*, *IQSEC2*, *AP3B2*) [10, 11, 24–45].

### Dystonia

Dystonia is a group of disorders characterized by abnormal movements and/or postures, with the following features: 1) sustained or intermittent muscle contractions causing repetitive abnormal movements and/or postures; 2) dystonia is usually patterned, twisting, and may be tremulous; 3) dystonia is often triggered or worsened by voluntary actions and may be associated with generalized muscle activation [46]. The etiology of dystonia is classified as genetic, acquired, or idiopathic. In patients with hemidystonia, structural imaging or neuropathological findings indicate that basal ganglia or its efferent pathways lesions are associated with contralateral dystonia [47]. Functional imaging studies provide evidence that dystonia is associated with abnormal activity in multiple brain regions, including the motor cortex, supplementary motor area, brainstem, cerebellum, and basal ganglia [48], suggesting that dystonia is a network disorder caused by dysfunction in one or more network nodes [49]. Electrophysiological and functional imaging studies indicate that the pathophysiology of dystonia may be associated with reduced central inhibition, increased plasticity, or impaired sensory function [49]. The underlying neurochemical mechanisms of dystonia are unclear; however, evidence suggests involvement of dopaminergic, cholinergic, GABAergic, and glutamatergic neurotransmitter systems [50]. Dystonia represents the most prevalent form of movement disorder observed in patients with genetic DEE. Dystonia in genetic DEE is highly associated with mutations in ion channel genes, including voltage-gated sodium channel genes (e.g., *SCN1A*, *SCN2A*, *SCN8A*) [11, 51–61], voltage-gated potassium channel genes (e.g., *KCNQ2*, *KCNT1*) [4, 11, 62, 63], and voltage-gated calcium channel genes (e.g., *CACNA1E*) [11, 64], ligand-gated ion channel GABA receptor genes (e.g., *GABRA1*, *GABRA5*, *GABRB2*) [65–67], and ligand-gated ion channel glutamate receptor genes (e.g., *GRIN2B*, *FRRS1L*, *GRIN1*) [68–70]. Additionally, dystonia is frequently associated with gene mutations affecting membrane transporter proteins (e.g., *SLC13A5*, *SLC1A2*, *SLC38A3*, *SLC32A1*, *ATP1A2*, *ATP1A3*) [11, 71]. In STXBP1-related DEE, dystonia represents the second most prevalent movement disorder.

### Chorea

Chorea may manifest in one of three ways: 1) as a series of randomly ordered, continuous, one or more discrete involuntary movements or movement fragments; 2) as continuous, slow, involuntary twisting movements that make it difficult for patients to maintain stable postures, known as slow chorea; 3) as chorea affecting proximal joints, such as the shoulders or hips [21]. This leads to large amplitude movements, which may exhibit flinging or waving characteristics. It is typical for normal infants to exhibit chorea-like movements, but this physiological chorea disappears by approximately eight months of age. A number of genetic disorders may result in the onset of chorea, such as Sydenham’s chorea, Huntington’s disease, and various types of spinocerebellar ataxia. Chorea is associated with the caudate nucleus, putamen, subthalamic nucleus, thalamus, and along with their interconnecting pathways. Damage or dysfunction in these structures disrupts the balance between the direct and indirect pathways of the basal ganglia circuit, leading to excessive dopaminergic activity and, ultimately, chorea [72]. In genetic DEE patients, chorea is often accompanied by dystonia. Mutations associated with chorea in DEE, as listed in the OMIM database, predominantly affect three categories of protein functions: ion channels (e.g., *CACNA1E*, *FRRS1L*, *GABRA1*, *GABRA2*, *GABRG2*, *GRIN1*, *GRIN2B*, *GRIN2D*, *KCNB1*, *KCNQ2*, *KCNT1*, *SCN1A*, *SCN2A*), protein transcription (e.g., *SLC13A5*, *SLC38A3*, *SLC32A1*), and enzymes/regulators (e.g., *ALG13*, *ARV1*, *CDKL5*, *FBX028*, *GNAO1*, *RHOBTB2*, *TBC1D24*, *UGDH*) [8, 10, 11, 62, 67–69, 73–75].

### Myoclonus

Myoclonus is a movement disorder characterized by brief, shock-like involuntary movements caused by muscle contraction or inhibition [21]. Muscle contractions result in positive myoclonus, while muscle inhibition causes negative myoclonus (e.g., asterixis). Patients typically describe myoclonus as “twitching”, “jerking”, or “spasms” [21]. Various causes, anatomical bases, and pathophysiological characteristics are involved in myoclonus [76]. It is classified based on etiology into four categories: physiological, primary, epileptic, and secondary. Based on the physiological mechanism of myoclonus, it can be classified into cortical, cortical-subcortical, subcortical-non-segmental, segmental, and peripheral myoclonus [77]. Currently, there is no unified mechanism for cortical myoclonus, and it may involve multiple mechanisms. The physiological mechanism of cortical-subcortical myoclonus is characterized by abnormalities in the bidirectional connections between the cortex and subcortex, with paroxysmal and excessive oscillations. Clinically, myoclonic seizures in epilepsy exemplify this phenomenon. A detailed exploration of these two types of myoclonus falls outside the scope of this section, as the myoclonus discussed here specifically refers to non-epileptic myoclonus. The physiological mechanisms of subcortical-non-segmental myoclonus, including reticular reflex myoclonus and spinal segmental myoclonus, are relatively well understood. These types of myoclonus involve abnormal electrical activity starting in local areas of the neural axis, and subsequently spreads bidirectionally, and involving bilateral pathways, leading to generalized myoclonus. Peripheral myoclonus results from lesions affecting the peripheral nervous system, leading to hyperactive motor discharges in the muscles it innervates [78]. A review of the OMIM database reveals that the majority of DEE-related mutated genes associated with myoclonus primarily involve ion channel protein functions, such as *CACNA1E*, *FRRS1L*, *GABRA1*, *GABRB2*, *GRIN1*, *KCNA2*, *KCNB1*, *KCNQ2*, *KCNT1*, *SCN1A*, *SCN2A*, and *SCN8A* [8, 10, 11, 69, 79–83].

### Ataxia

Ataxia refers to the disruption of the smooth, and precise coordination of movements, which most commonly presents as an instability in gait. In children, it may simply present as a refusal to walk. Ataxia is typically the result of cerebellar dysfunction. However, disturbances at various levels of the frontal lobe, inner ear, vestibular nerve, and proprioceptive systems (including peripheral nerves and spinal cord) can also affect coordination, leading to ataxia [84]. According to the disease course, ataxia can be categorized as acute, episodic, chronic progressive, or chronic non-progressive. Among these, episodic, chronic progressive, and chronic non-progressive ataxia types are often associated with genetic disorders. Ataxia may manifest either during or subsequent to an epileptic seizure [84]. Non-convulsive seizures (without changes in consciousness or the presence of abnormal movements) may present solely as ataxia [85]. Mutations in genes encoding ion channels, ion pumps, and glutamate transporters have been linked to the development of episodic ataxia and epileptic manifestations [86, 87]. These symptoms may be attributed to neuronal excitability dysfunction, which is a potential mechanism. In genetic DEE, patients with ataxia often exhibit additional movement disorders. In STXBP1-related DEE, ataxia is the most common movement disorder and can occur alone or in combination with other movement disorders, including dystonia, tremor, or chorea. Among the DEE-related mutated genes listed in the OMIM database, ataxia is mainly observed in mutations affecting the following two major categories of protein function: ion channels (e.g., *CACNA1A*, *GABRB1*, *GABRB2*, *GABRB3*, *GABRG2*, *KCNA2*, *KCNC2*, *KCNQ2*, *SCN1A*, *SCN1B*, *SCN2A*, *SCN8A*) and enzymes/regulatory proteins (e.g., *ALG13*, *ARHGEF9*, *ARV1*, *CDKL5*, *FBX028*, *PIGS*) [8, 10, 11, 74].

### Tremor

Tremor is an involuntary, rhythmic movement of a part of the body [88]. While the frequency may be relatively constant in some patients, the amplitude may vary [21]. Tremor is caused by the alternating or synchronous contraction of antagonist muscles [89]. Tremor can be classified as resting or action tremor depending on whether it is induced by factors, and as isolated or combined tremor depending on whether it is accompanied by neurological and/or other systemic symptoms and signs. The mechanism of tremor may involve lesions affecting the cortico-striato-thalamo-cortical loops that integrate complex muscle group movements, and the red nucleus, inferior olivary nucleus, and dentate nucleus regions that regulate autonomic subtle movements [90]. Tremor is the most common type of movement disorder, and many normal individuals may experience exaggerated physiological tremor [91]. Various diseases, drugs, toxins, and substances can cause tremor, which can be broadly categorized as genetic, acquired, or idiopathic. Among DEE-related mutated genes listed in the OMIM database, tremor as a movement disorder is relatively rare, and involves multiple pathways, including ion channels (e.g., *CACNA1A*, *KCNQ2*, *SCN2A*, *SCN8A*, *SCN1A*), enzymes/regulatory proteins (e.g., *CDKL5*, *CHD2*, *UGDH*, *DNM1*), membrane transport regulation (e.g., *NAPB*, *STXBP1*), nucleic acid-binding proteins (e.g., *CELF2*), and cellular metabolism and signal transduction (e.g., *YWHAG*) [8, 10, 11, 26, 29].

### Hypokinesia

Hypokinetic movement disorders are frequently accompanied by muscle rigidity, postural instability, and a reduction in spontaneous movement [21]. The basal ganglia are a set of deep nuclear complexes, that include the striatum, globus pallidus, and substantia nigra, with the substantia nigra pars compacta forming the nigrostriatal pathway as the main dopaminergic pathway. Lesions in this pathway are associated with hypokinetic movement disorders. The basal ganglia play a crucial role in regulating the initiation, strength, and control of movements. Biochemical or structural abnormalities within these structures can result in the onset of movement disorders. A number of genetic disorders have been identified as potential causes of hypokinesia, such as Wilson’s disease, juvenile Huntington’s disease, and neurodegeneration with brain iron accumulation. Currently, reports of DEE associated with hypokinesia are relatively rare. A review of the OMIM database revealed that mutations in several genes are associated with hypokinesia, include *ATP1A3*, *PIGP*, *SCN2A*, *SCN8A*, *TBC1D24*, and *WWOX* [8, 10, 11].

As of 1 August, 2024, 118 DEE-related mutation genes have been recorded in the OMIM database. The most commonly mutated genes associated with movement disorders in DEE are shown in Table 1. Among various movement disorder phenotypes, dystonia is the most prevalent, followed by ataxia, chorea, myoclonus, stereotypies, tremors, and hypokinesia, which are less common. The mutated genes are involved in a range of protein functions, including ion channels, enzymes/regulatory proteins, membrane transporters, cellular metabolism and signal transduction, and nucleic acid-binding proteins.

**Table 1:** Mutation Genes Related to DEE with Movement Disorders (Recorded in OMIM)

Movement disorders	Related types of DEE/Associated mutant genes
Stereotypies	DEE2, 4, 7, 6B, 11, 16, 17, 19, 31A, 36, 42, 43, 48, 58, 64, 66, 67, 74, 84, 85, 94, 98, 99, 101, 104, 107, 108
	*ALG13, AP3B2, ATP1A2, ATP1A3, ATP6V0A1, CACNA1A, CDKL5, CHD2, CUX2, DNM1, GABRA1, GABRB3, GABRG2, GNAO1, GRIN1, KCNQ2, MAST3, NAPB, NTRK2, PACS2, RHOBTB2, SCN1A, SCN2A, SMC1A, STXBP1, TBC1D24, UGDH,*
Dystonia	DEE1, 2, 4, 5, 6B, 7, 11, 13, 14, 17, 19, 25, 26, 27, 28, 31A, 32, 36, 37, 38, 40, 41, 42, 44, 48, 51, 53, 55, 58, 64, 68, 69, 76, 79, 84, 89, 92, 99, 100, 101, 102, 106, 114
	*ACTL6B, ALG13, AP3B2, ARV1, ARX, ATP1A2, ATP1A3, CACNA1A, CACNA1E, CDKL5, DNM1, FBX028, FRRS1L, GABRA1, GABRA5, GABRB2, GAD1, GNAO1, GRIN1, GRIN2B, GUF1, KCNB1, KCNA2, KCNQ2, KCNT1, MDH2, NTRK2, PIGP, RHOBTB2, SCN1A, SCN2A, SCN8A, SLC13A5, SLC1A2, SLC32A1, SLC38A3, SPTAN1, STXBP1, SYNJ1, TRAK1, UBA5, UFSP2, UGDH, WWOX*
Chorea	DEE1, 2, 4, 6B, 7, 11, 13, 14, 16, 17, 19, 25, 26, 27, 36, 37, 38, 42, 46, 64, 69, 74, 78, 84, 100, 101, 102, 114
	*ALG13, ARV1, ARX, CACNA1A, CACNA1E, CDKL5, FBX028, FRRS1L, GABRA1, GABRA2, GABRG2, GNAO1, GRIN1, GRIN2B, GRIN2D, KCNB1, KCNQ2, KCNT1, RHOBTB2, SCN1A, SCN2A, SCN8A, SLC13A5, SLC32A1, SLC38A3, STXBP1, TBC1D24, UGDH*
Myoclonus	DEE6B, 7, 14, 16, 17, 26, 28, 37, 40, 42, 43, 49, 55, 68, 92, 100, 102, 104
	*CACNA1E, CDKL5, FRRS1L, GABRA1, GABRB2, GNAO1, GRIN1, KCNA2, KCNB1, KCNQ2, KCNT1, SCN1A, SCN2A, SCN8A,, STXBP1, TBC1D24, UBA5, WWOX*
Ataxia	DEE2, 4, 5, 6B, 7, 8, 11, 13, 16, 17, 24, 25, 28, 32, 36, 38, 42, 43, 45, 52, 56, 64, 74, 92, 95, 99, 100, 103, 104
	*ALG13, ARHGEF9, ARV1, ATP1A3, ATP6V0A1, CACNA1A, CDKL5, FBX028, GABRB1, GABRB2, GABRB3, GABRG2, GNAO1, HCN1, KCNA2, KCNC2, KCNQ2, PIGS, RHOBTB2, SCN1A, SCN1B, SCN2A, SCN8A, SLC13A5, SPTAN1, STXBP1, TBC1D24, WWOX, YWHAG*
Tremor	DEE2, 4, 6B, 7, 11, 13, 17, 28, 31A, 32, 42, 56, 84, 94, 97, 107
	*CACNA1A, CDKL5, CELF2, CHD2, DNM1, GNAO1, KCNA2, KCNQ2, NAPB, SCN1A, SCN2A, SCN8A, STXBP1, UGDH, WWOX, YWHAG*
Hypokinesia	DEE4, 7, 11, 13, 16, 28, 42, 55, 99, 103
	*ATP1A3, CACNA1A, SCN2A, SCN8A, STXBP1, TBC1D24, WWOX, PIGP, KCNC2, KCNQ2*

## Common single-gene mutations associated with DEE and movement disorders

In order to further understand the distribution characteristics of movement disorders in common single-gene mutations associated with DEE, we conducted a co-occurrence analysis of keywords.

### Literature search

A search of the Web of Science Core Collection was conducted across all databases within the Web of Science for articles related to the topic of “Genetic DEE with Movement Disorders Research Advances”. The search covered research areas that included clinical medicine, neuroscience, molecular genetics, and pediatrics, and covered literature types included papers, reviews, and case reports. The search was conducted across the entire time span and took place in July 2024. The records were exported in plain text format, including the complete records and cited references (Figure S1).

### Literature inclusion and exclusion criteria

Inclusion criteria:

1. Articles containing Medical Subject Headings (MeSH) terms related to DEE (e.g., Developmental and Epileptic Encephalopathy) and movement disorders (e.g., Movement Disorders OR Stereotypies OR Dystonia OR Chorea OR Ataxia OR Myoclonus OR Hypokinesia OR Tremor);
2. Publication time range encompassing all years;
3. Only research papers, case reports, and reviews were included.

Exclusion criteria:

1. Duplicate studies;
2. Articles not related to genetic DEE with movement disorders, i.e., excluding literature on DEE or movement disorders alone.

### Visualization analysis

**Fig. 1:**
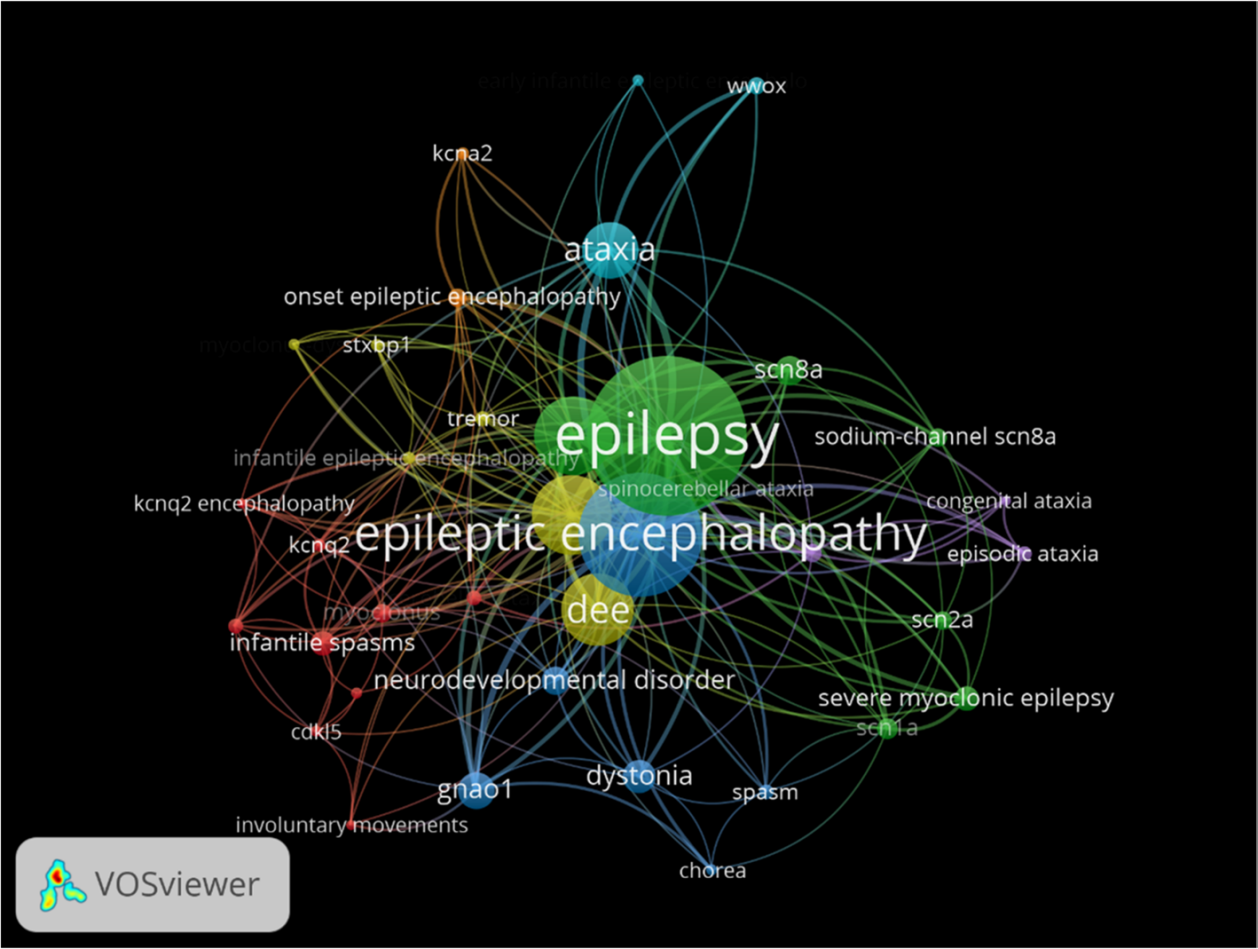
Co-occurrence analysis of genetic DEE and movement disorders related keywords by VOSviewer. The various circles represent different genes or phenotypes, with larger circles indicating a higher frequency of occurrence. The lines connecting the circles indicate the existence of associations, with thicker lines representing stronger association

VOSviewer software was employed for the generation of visual maps, specifically keyword visualization analysis. Initially, the top ten genes related to genetic DEE with movement disorders were selected based on word frequency. Subsequently, a co-occurrence analysis was conducted to examine the relationships between these genes and high-frequency movement disorder-related keywords. The resulting visual analysis demonstrates the types of movement disorders that are closely associated with each gene and the genes closely associated with each movement disorder type (Fig. 1).

### VOSviewer software keyword co-occurrence analysis results

As shown in Fig. 1, our keyword co-occurrence analysis using VOSviewer software identified the ten most significant genes associated with single-gene DEE with movement disorders. The identified genes were *GNAO1*, *SCN8A*, *CACNA1A*, *SCN1A*, *WWOX*, *CDKL5*, *SCN2A*, *KCNQ2*, *STXBP1*, and *KCNA2*. The spectrum of movement disorders include involuntary movements, dystonia, ataxia, chorea, myoclonus, tremors, spasms, and central motor disorders.

### Characteristics of movement disorders associated with the top ten DEE-related genes

To further understand the characteristics of movement disorders associated with the ten most frequently occurring DEE-related genes identified through VOSviewer software's keyword co-occurrence analysis, we conducted a further statistical analysis of the types of movement disorders related to these genes. The methods are as follows:

1. The search databases, research areas, time span, and search time are identical to those used in “Literature search” section;
2. The search terms were “developmental and epileptic encephalopathy” [All Fields] AND “GNAO1 OR SCN8A OR CACNA1A OR SCN1A OR WWOX OR CDKL5 OR SCN2A OR KCNQ2 OR STXBP1 OR KCNA2” [All Fields].
3. The types of movement disorders associated with different mutated genes were classified and statistically analyzed in the relevant original research literature. Only articles that provided specific descriptions of movement disorder types and their corresponding proportions were included in the statistical table (Table 2). The number of cases for some genes was limited due to the lack of clarity regarding the descriptions of movement disorder types and the specific case numbers in some previous reports.

**Table 2:** Characteristics of movement disorders associated with the top ten DEE-related genes

Gene	Stereotypies	Dystonia	Chorea	Ataxia	Myoclonus	Tremor	Hypokinesia
*GNAO1*	6/58	38/58	39/58	2/58	3/58	3/58	0
*CDKL5*	42/53	7/53	15/53	3/53	2/53	4/53	0
*SCN1A*	9/39	9/39	6/39	5/39	24/39	6/39	0
*CACNA1A*	1/29	10/29	2/29	8/29	0	16/29	12/29
*KCNQ2*	6/24	15/24	3/24	1/24	4/24	1/24	4/24
*SCN2A*	4/22	10/22	4/22	2/22	3/22	1/22	1/22
*STXBP1*	10/21	8/21	3/21	1/21	5/21	2/21	3/21
*SCN8A*	0	9/20	2/20	1/20	12/20	4/20	1/20
*WWOX*	0	15/18	0	2/18	1/18	1/18	5/18
*KCNA2*	0	2/4	0	1/4	3/4	0	0
4. Among the ten most commonly DEE-associated movement disorder genes, stereotypies are most frequently observed in patients with the *CDKL5* gene mutations (approximately 79.2%), and are also notably observed in patients with *KCNQ2* and *STXBP1* mutation-related DEE patients. Dystonia is associated with all ten genes, being the main type of movement disorder in *GNAO1* (approximately 64.4%), *KCNQ2* (62.5%), and *WWOX* (83.3%) mutations. Chorea is primarily associated with the *GNAO1* gene (approximately 67.2%). Myoclonus is the main type of movement disorder associated with *CACNA1A* (approximately 82.8%), *KCNA2* (75%), *SCN1A* (approximately 61.5%), and *SCN8A* (60%) mutations. Furthermore, tremor is a prevalent movement disorder in *CACNA1A* mutation-related DEE patients, occurring in approximately 55.2% of cases.

## Treatment of movement disorders

DEE patients often have pharmacoresistant epilepsy [1], and in our clinical practice, treatment has primarily focused on controlling seizures, with less attention given to treating movement disorders. Despite the unfavorable treatment outcomes for movement disorders in these patients [11], the management of movement disorders, especially dystonia and chorea, remains important. The appropriate treatment of movement disorders depends on accurate diagnosis [92], and it is primarily symptomatic. The treatment of dystonia encompasses a range of pharmacological and non-pharmacological interventions. Oral medications, such as levodopa, benzhexol, baclofen, and benzodiazepines, represent a key aspect of this approach. Additionally, botulinum toxin injections, and deep brain stimulation are also utilized in the management of dystonia. In the case of chorea, dopamine receptor blockers are generally considered the most effective medications for reducing choreiform movements. Second-generation (atypical) antipsychotics, such as olanzapine, risperidone, and aripiprazole, are commonly used to alleviate chorea. Additionally, dopamine-depleting drugs, such as tetrabenazine, valbenazine, and deutetrabenazine, may also be effective. Antiseizure medications, such as valproate, carbamazepine, oxcarbazepine, topiramate, and levetiracetam, may help suppress chorea in some patients [93]. In a series of genetic DEE cases with movement disorders reported by van der Veen et al. [11], 46% (34/74) of patients received treatment for movement disorders. Among the patients who were followed, tetrabenazine improved dystonia and chorea caused by *GNAO1* and *UGDH* mutations but was ineffective in three patients with different sodium channel mutations (*SCN1A*, *SCN2A*, and *SCN8A*). Two patients with paroxysmal dystonia showed some improvement with tetrabenazine in combination with other medications (baclofen, clonazepam, and lorazepam). Three patients with dystonia did not respond to levodopa treatment. One patient with *SCN2A* mutation-related DEE showed improvement in dystonia after intrathecal baclofen injection. Another DEE patient with *GNAO1* mutation showed improvement in chorea and dystonia after deep brain stimulation surgery in the bilateral globus pallidus internus [89].

## Conclusions and future prospects

Movement disorders have a significant impact on the quality of life in DEE patients and require integrated management of seizures, developmental disorders, and movement disorders. Accurate classification and description of movement disorders will benefit the comprehensive management of DEE patients. Detailed clinical characterization will aid in the interpretation and analysis of genetic data. As next-generation sequencing becomes more widely used in clinical practice, the genetic spectrum of genetic DEE with movement disorders will continue to expand. Accurate interpretation of genetic test results will assist in disease prediction and genetic counseling, determine whether specific management strategies for genetic defects should be employed in treatment, and guide the development of more precise medical treatments in the future.

## Supplementary Information

Supplementary Material 1.

## Data Availability

Availability of data and materials is not applicable in this study.

## Abbreviations

DEE: Developmental and epileptic encephalopathies
EEG: Electroencephalogram
ILAE: International League Against Epilepsy
MeSH: Medical Subject Headings
OMIM: The Online Mendelian Inheritance in Man
