# Supplementary material for: Advances in genetic developmental and epileptic encephalopathies with movement disorders
Source: Acta Epileptol. 2025 Feb 3;7:9. doi: 10.1186/s42494-024-00194-z (PMC11960234; doi:10.1186/s42494-024-00194-z)
Supplement: Supplementary file 1 — Supplementary Material 1. [file 42494_2024_194_MOESM1_ESM.docx]

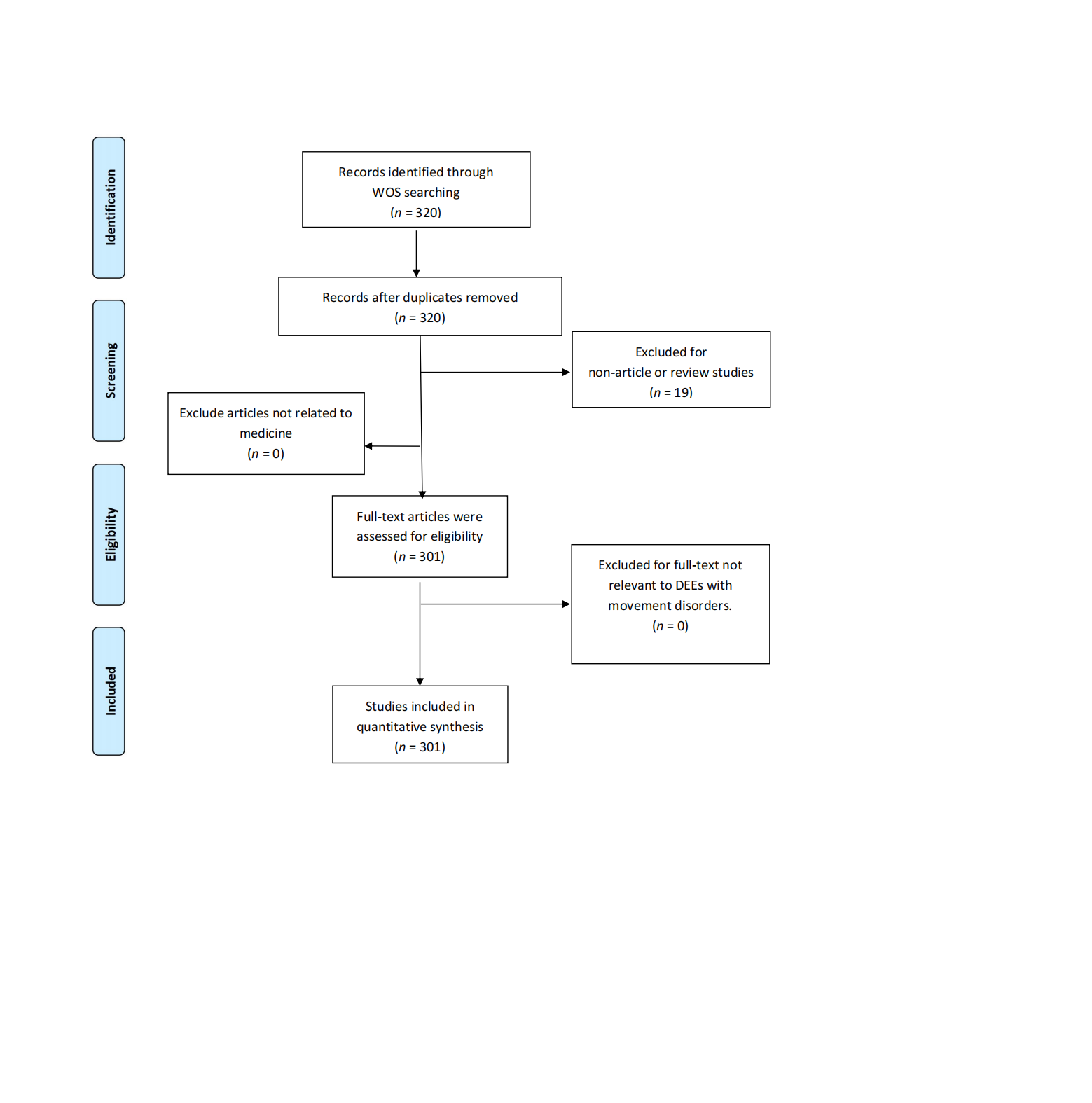


**Figure S1:** Flowchart of literature screening for “Genetic DEE with Movement Disorders Research Advances”
